# Sociocultural determinants of antimicrobial resistance in Iran: a qualitative study

**DOI:** 10.1186/s12889-025-23361-4

**Published:** 2025-07-10

**Authors:** Mojtaba Mehtarpour, Zahra Najafi, Ebrahim Jaafaripooyan

**Affiliations:** 1 Social Determinants in Health Promotion Research Center, Hormozgan Health Institute, Hormozgan University of Medical Sciences, Bandar Abbas, Iran; 2 East Hormozgan School of Medical Sciences, Minab, Iran; 3 Department of Health Management, Policy and Economics, School of Public Health, Tehran University of Medical Sciences, Tehran, Iran

**Keywords:** Sociocultural factors, Antimicrobial resistance, Antimicrobial overuse, Qualitative research, Iran

## Abstract

**Introduction:**

Antimicrobial resistance (AMR) is a growing global public health threat that is highly likely to undermine modern medicine and pose significant risks to health systems and national economies worldwide. As such, its complex nature, as affected by biological, environmental, economic, and sociocultural factors, requires a multidisciplinary approach to its control. Therefore, uncovering the sociocultural drivers of AMR is critical to designing effective interventions, particularly in low- and middle-income countries.

**Methods:**

This qualitative study included a number of 57 semi-structured, face-to-face interviews with policymakers, managers, and service providers from both human and animal health sectors using purposive and snowball sampling techniques. The data were subsequently analyzed employing thematic analysis.

**Findings:**

Several sociocultural factors were uncovered contributing to the development of AMR, in relation mainly with general public culture, public drug use culture, cultural barriers among service providers, and demographic changes in the country.

**Conclusion:**

Sociocultural factors within both human and animal health sectors might significantly influence the antimicrobials usage and the proliferation of AMR across diverse ecosystems, underscoring the imperative for policymakers to consider them when devising interventions to combat AMR. Policymakers are advised to prioritize the development of clinical guidelines, enhance insurance oversight, and improve diagnostic capabilities in an effort to effectively address the challenge of AMR.

**Supplementary Information:**

The online version contains supplementary material available at 10.1186/s12889-025-23361-4.

## Introduction

Antimicrobial resistance (AMR) is increasingly being recognized as a serious global health threat. Recent estimates indicate that bacterial AMR was directly responsible for 1.14 million deaths and associated with 4.71 million deaths worldwide in 2021 [1]. This growing phenomenon seems to have undermined the achievements of modern medicine and has prompted many countries to assume an urgent action. The prospect of a post-antibiotic era—where common infections and minor injuries could once again become fatal—is no longer a dystopian vision but a realistic concern of the 21st century [2]. According to the World Bank, AMR could reduce the gross domestic product (GDP) of low-income countries by over 5%, potentially pushing 28 million people—primarily in developing nations—into poverty by 2050 [3]. Without significant interventions, AMR is projected to cause up to 10 million deaths annually by 2050, with an associated economic and human cost estimated at $100 trillion [4].

The World Health Organization (WHO), in its Global Action Plan on AMR, emphasizes the importance of adopting a One Health approach and fostering collaboration across all sectors and disciplines [5]. However, countries within the Eastern Mediterranean Region, including Iran, encounter significant challenges in controlling AMR. These challenges include a lack of coordination and integration among various sectors, limited resources, a fragmented healthcare system, inadequate surveillance, and gaps in knowledge and health literacy among the population [6]. National data indicate that Iran is among the countries reporting high levels of multidrug resistance to seven bacterial pathogens of greatest international concern [2]. Antibiotic use in Iran has experienced a substantial surge, increasing from 33.6 defined daily doses (DDD) per 1,000 population per day in 2000 to 67.6 DDD in 2022 [7]. Notably, third-generation Cephalosporins have emerged as a dominant class, accounting for over 43% of total antibiotic use. Significant rises were also documented in the consumption of macrolides, beta-lactam/beta-lactamase inhibitor combinations, and fluoroquinolones, which were responsible for 58.6%, 56.8%, and 39.2% of the overall increase, respectively [8]. Furthermore, a study of outpatient practices found that nearly 43% of antibiotic prescriptions were deemed irrational [9].

Over the years, Iran has undertaken several initiatives to combat AMR; including the establishment of the National Committee for Rational Drug Use, the formation of hospital infection control committees, the development of a hospital infection surveillance system, the drafting of a national action plan to control AMR, and the implementation of various public awareness campaigns [6, 10]. The development and dissemination of AMR across different ecosystems —human, animal, and environmental— are shaped by a complex interplay of environmental, biological, technological, economic, and sociocultural factors. Without a comprehensive understanding of the sociocultural context—including public beliefs and the dynamics of patient-provider relationships—efforts to control AMR are unlikely to succeed [11].

The overuse, misuse, and underuse of antimicrobials are major contributors to the emergence of AMR [4, 5, 12]. In Iran, several barriers to appropriate antimicrobial prescribing have been identified; including the low professional competence among physicians, limited access to information resources and clinical guidelines, weak oversight of rational prescribing practices, and an unfavorable context for rational antimicrobial use. Additionally, sociocultural factors—such as the public misconceptions about antimicrobials, patient pressure for unnecessary prescriptions, and the over-the-counter availability—further exacerbate inappropriate prescribing [13].

Additional sociocultural factors influencing irrational antimicrobials use in Iran include a tendency toward self-medication, low patient awareness regarding the dangers associated with irrational antimicrobial use, improper antimicrobials consumption behaviors (such as altering dosages, self-initiated discontinuation, or changing the timing of medication), the influence of recommendations from peers or family members on antimicrobials use, and the excessive consumption of herbal and traditional medicines [14]. Behavioral patterns across all stakeholder groups—including the public, healthcare providers, and livestock breeders—significantly contribute to the development of AMR. Many aspects of AMR are behaviorally driven and must be examined within each country’s unique sociocultural context [15]. While much of the existing literature on AMR in Iran has focused either on clinical patterns of resistance [16, 17] or on the perspectives of limited professional groups [13, 18, 19], very few studies have systematically explored the broader sociocultural determinants of this issue. This study aims thus to fill this gap by providing an in-depth analysis of the sociocultural drivers of AMR in Iran.

## Methods

### Study design

This was a qualitative exploratory study designed to identify and understand the sociocultural factors influencing AMR in Iran.

### Sample

Participants were selected using purposive and snowball sampling techniques. Participants were recruited based on at least one of the following inclusion criteria: relevant knowledge about AMR, professional experience in the field, involvement in the antimicrobial restriction policy at the national level, and willingness to participate in the study. Participants were invited based on the inclusion criteria, and all participants took part voluntarily. Interviews continued until data saturation was reached, indicating that no new themes emerged. Fifty-seven individuals were interviewed (36 men and 21 women), with an average of 18 years of professional experience (Table 1). One eligible individual declined to participate after receiving the study information and consent form.

**Table 1:** Participants

Number	Participants	Count
1–6	Deputy of Public Health	6
7–12	Food and Drug Administration	6
13–15	Deputy of Treatment	3
16	National Reference Laboratory of the Ministry of Health	1
17	Health Insurance Organization	1
18–27	Veterinary Organization	10
28–29	Veterinary Council	2
30	Supreme Council for Health and Food Security	1
31	Pilot Project Manager for Antimicrobial Resistance Surveillance	1
32–35	Infectious Disease Specialists Association	4
36–37	Specialized Microbiology Associations	2
38–42	Infectious Disease Specialists	5
43	Veterinarian	1
44	Health Commission of the Islamic Parliament of Iran	1
45	Standards Organization	1
46	Medical Council	1
47–52	Researchers in Antimicrobial Resistance (Human and Animal Health)	6
53–54	Health Policy Specialists	2
55–56	Food Safety Specialists	2
57	Livestock Breeder	1

### Data collection

Data were collected through semi-structured, face-to-face interviews conducted at the participants’ workplaces, to ensure participant comfort and convenience. Prior to the interviews, participants received an information sheet and informed consent form via email. The interview guide was developed based on the study objectives and a review of relevant literature. It included open-ended questions exploring participants’ views on factors contributing to AMR, barriers to effective antibiotic stewardship, and the influence of cultural, social, and systemic factors. Five pilot interviews were conducted to refine the guide and enhance its clarity and relevance. Interviews were conducted by MM; most were audio-recorded with participants’ consent and transcribed verbatim. In cases where recording was not possible, detailed notes were taken. Two participants were interviewed twice, resulting in a total of 59 interviews, with an average duration of 55 min. To minimize the self-report and interviewer bias, several strategies were applied. For instance; the interview questions were designed to be open-ended and neutrally worded to reduce the influence of interviewer expectations. Participants were fully assured of confidentiality and anonymity, which helped encourage honest and open responses. The interviewer (MM) maintained a neutral and nonjudgmental stance during interviews and took field notes to capture contextual factors. In addition, responses were triangulated across participants from different sectors and professional roles to reduce reliance on individual narratives.

### Data analysis

Data were analyzed using thematic analysis, following Braun and Clarke’s six-phase approach [20]. This inductive and data-driven method was well-suited to the exploratory nature of the study. The analysis began with familiarization through repeated reading of interview transcripts and field notes. Manual, iterative coding was then conducted to identify meaningful units of data. These codes were examined and grouped into initial themes, which were further refined, clearly defined, and organized into a coherent thematic structure reflecting the sociocultural influences on AMR in Iran. MAXQDA 2018 software was used to support data management and coding. Thematic categories were revisited throughout the process to ensure they accurately captured the participants’ perspectives.

To ensure trustworthiness, we applied several strategies based on the Lincoln and Guba’s [21] criteria. Credibility was supported through maximum variation sampling, prolonged engagement, and participant validation of preliminary findings. Transferability supported by providing rich contextual and demographic details. Dependability was ensured through systematic documentation of the research process, while confirmability was strengthened by maintaining an audit trail and discussing analytic decisions within the research team. Triangulation across diverse professional roles and sectors (including both human and animal health) added depth and minimized potential bias. The study adhered to the COREQ (Consolidated Criteria for Reporting Qualitative Research) checklist, which is included as a supplementary file.

### Findings

The sociocultural factors underling AMR were categorized into five main themes: general public culture, public drug use culture, service providers’ culture, awareness and knowledge among livestock farmers, and demographic changes (Table 2).

**Table 2:** Sociocultural determinants of AMR in Iran

Theme	Sub-theme
General Public Culture	• Lack of Effective Public Education Policies • Behavioral Tendencies Towards Quick and Ineffective Treatments • Skepticism Towards Industrial Products • Demand for Heavier Chickens • Lack of Public Awareness of Patient Rights
Public Drug Use Culture	• Pressure on Service Providers to Prescribe Antibiotics • Pressure from Peers for Irrational Use • Misconceptions About Antibiotics • Incomplete Adherence to Treatment Regimens • Negative Influence of Social Media • Unregulated Access and Storage of Antibiotics • Lack of Commitment to Timely Medication
Service Providers Culture	• Weak Teamwork in Service Delivery • Physicians’ Fear of Bad Reputation and Over-Prescribing • Negative Impact of Medical Authority on Adherence to Guidelines • Lack of Professional Ethics • Lack of Positive Attitude Toward Hand Hygiene • Unawareness of the Problem by Veterinarians • Perception of Veterinary Antimicrobials as Less Important than Human Antimicrobials • Physicians’ Preference for New Antimicrobials • Neglect of Sexually Transmitted Diseases by Policymakers
Livestock Farmers’ knowledge	• Low literacy levels among livestock farmers • Poor awareness about medications
Demographic Changes	• The Role of Development and Population Growth in Increased Antimicrobial Consumption • Impact of Population Aging • Urban Marginalization and Limited Access to Adequate Services

## General public culture

The findings highlight that general public culture plays a pivotal role in the irrational use of antimicrobials and the exacerbation of AMR. A lack of effective public education programs, Behavioral Tendencies towards Quick and Ineffective Treatments, and skepticism towards industrial products all contribute to behaviors that exacerbate AMR. Many individuals rely on self-medication, traditional treatments, and mistrust scientific interventions, while others demand products like heavier chickens, that increase the use of antibiotics in food production. Additionally, public misconceptions about patient rights further fuel the cycle of AMR. These cultural factors create an environment where irrational use of antimicrobials becomes normalized, underscoring the need for comprehensive public health intervention.

### Lack of effective public education policies

Interviewees expressed significant concerns regarding the lack of targeted and ongoing public education programs addressing AMR, the rational use of antimicrobials, and the characteristics of healthy food. They noted that existing programs have not effectively engaged other organizations, such as media outlets or municipalities, due to both qualitative and quantitative shortcomings. One participant commented:

*“The media demands payment for broadcasting information. Their programs are more focused on treatment rather than prevention and awareness. How often do you see the media producing and airing quality content on AMR in popular programs?” (P.10).*

### Behavioral tendencies towards quick and ineffective treatments

Some patients believe that rapid recovery requires the use of antibiotics, often dismissing recommendations for rest or non-pharmacological treatments. They also tend to show impatience toward essential diagnostic procedures, including antibiotic susceptibility testing. An Iranian Health Insurance Organization manager explained: *“Why isn’t an antibiogram done? Patients are impatient. If you send them to the lab*,* it takes time*,* and they think the lab is colluding with the doctors. They believe the process takes unnecessarily long” (P.17).*

The widespread use of unscientific treatment methods often leads to delays in receiving appropriate care. Recently, growing skepticism toward evidence-based preventive strategies—such as vaccination—has been observed. According to participants, some individuals and even professionals perceive such interventions as part of broader conspiracies. One physician noted: *“Even educated doctors exaggerate and claim that these drugs and vaccines are brought by enemies to harm our people. Such beliefs arise due to political and religious issues”* (P.33). Another physician commented on the misuse of traditional medicine: *“It has turned into charlatanism in our country. I had a patient who consulted such practitioners*,* and by the time he came to me*,* his disease had progressed”* (P.40). Furthermore, self-medication with antibiotics was reported as relatively common, partly driven by individuals’ reluctance to pay for medical consultations (P.9).

### Skepticism towards industrial products

Skepticism towards industrially produced food has contributed to the growing popularity of traditional food products. However, these traditional products are often sold outside the regulatory framework of relevant authorities, increasing the risk of consuming food containing antibiotic residues. One participant noted,

*“The culture of consuming pasteurized milk hasn’t caught on in our country. There is a belief that anything nostalgic is healthier. Some people think pasteurized milk is full of chemicals to preserve it for months” (P.29).*

### Demand for heavier chickens

Due to low awareness, heavier chickens are more popular in the market, leading to increased demand that encourages producers to raise chickens on farms for longer periods. This extended farming duration elevates the risk of disease and the consequent use of antimicrobials, which in turn raises the likelihood of antimicrobials residues in food products. One participant explained:

*“People usually want chickens over two kilograms. The producer has to keep them for over 45 days*,* increasing the risk of diseases. Therefore*,* they use antibiotics in the last days as prophylaxis or treatment*,* adding it to their water and food until the last night before sending them to the slaughterhouse” (P.55).*

### Lack of public awareness of patient rights

When patients are unaware of their rights, they tend to remain a silent group with little demand for improved infection control and the rational use of antimicrobials. Interviewees expressed the belief that if a patient contracts a hospital-acquired infection, they are unlikely to pursue complaints or advocate for improvements in infection control and prevention. One participant commented:

*“Patients often don’t know their rights or those of their caregivers. If an infection occurs*,* there’s little follow-up*,* and patients don’t advocate for better infection prevention and control*,* believing it’s just part of hospital life” (P.49).*

## Public drug use culture

The main theme of “Public Drug Use Culture” reflects societal factors that contribute to the irrational use of antibiotics. This includes external pressures on healthcare providers to prescribe antibiotics, both from patients and food producers, who often demand these drugs despite their limited necessity. Peer influence also plays a significant role, with non-specialists offering advice that can lead to improper use, whether by promoting unnecessary medication or discouraging needed treatment. Additionally, misconceptions about antibiotics, misinformation from social media, and behaviors such as storing medications at home contribute to the culture of misuse. These practices, combined with a lack of adherence to prescribed treatment regimens and easy access to antibiotics, exacerbate the problem of AMR.

### Pressure on service providers to prescribe antibiotics

Interviewees from both the human and animal health sectors indicated that individuals and food producers frequently make irrational requests for antimicrobials, exerting pressure on service providers such as physicians, pharmacists, and veterinarians to comply with these demands. A member of the National Committee for Rational Drug Use described the situation, stating:

*“Patients are insistent on getting antibiotics*,* even if they just have a cold. We say it’s not necessary*,* but they go to the pharmacy and insist on getting it without a prescription” (P.9).* Similarly, a manager from the Veterinary Organization highlighted a recurring issue in animal health, remarking:

*“Unfortunately*,* clients believe that a veterinarian who prescribes more antibiotics is better. They are not interested in hearing about hygiene measures or preventive guidelines. If the veterinarian says a certain antimicrobials is not needed*,* they insist on obtaining it anyway” (P.19).*

### Pressure from peers for irrational use

Recommendations from non-experts regarding the use or non-use of antimicrobials were frequently highlighted in the interviews. One participant remarked: *“Non-specialist opinions are rampant. If I improved with an antibiotic*,* I prescribe it to others as well. Our view of antibiotics is like candy” (P.48).*

In some instances where a patient should use an antimicrobial, inappropriate advice can lead to its non-use, exemplifying another form of irrational consumption. An infectious disease specialist explained: *“Sometimes people swing to the other extreme. We prescribe medication and inform them that they have a urinary infection and need to take antibiotics*,* but then their relatives*,* like aunts and uncles*,* say that antibiotics are harmful to the child and they shouldn’t use them. After a few days*,* we see the problem has worsened” (P.39).*

### Misconceptions about antibiotics

Interviewees in this study identified several misconceptions that contribute to the irrational use of antimicrobials (Table 3).

**Table 3:** Misconceptions about antibiotics

Misconception	Sample Quotation (Participant Number) (Organizational Affiliation)
Antibiotics are safe and strengthen the body against microbes	People often don’t see immediate and severe side effects from the drug, which leads to a lack of awareness about its consequences, resulting in reckless usage. They believe it doesn’t harm and think it kills all the microbes. (P.3), (Health Ministry Administrator)
Injectable antibiotics are always preferable	Many people think that using injectable antibiotics is better than oral forms. They often request their doctors to prescribe these antibiotics and, if the doctor refuses, they consider them less competent and may not visit them again. (P.9), (Administrator from the Rational Drug Use Committee)
Antibiotics are effective for colds and allergies	Someone catches a cold and says, ‘I won’t get better until I take an injection.’ It’s a mistaken belief that for a viral cold, one needs antibiotics. Even if the doctor hasn’t prescribed it, they’ll find a way to get it. (P.33), (Infectious Diseases Society Administrator
Specific antibiotics can treat all diseases	A patient says, ‘Whenever I get an infection, I must take the 500 mg capsule.’ Whether it’s an ear infection, sore throat, a wound on the leg, or diarrhea, they take the same thing. They have a strong belief in it. Whether it’s ampicillin or amoxicillin” (P.33), (Infectious Diseases Society Administrator)
Insistence on prescribing newer and more expensive drugs as more effective	A person comes in with strep throat or a urinary tract infection, which could be treated with a simple first-line antibiotic. They believe that if you give them an antibiotic that costs 100,000 Toman, it’s more effective. Or they suggest the newest drug that just hit the market” (P.51), (Infectious Disease Specialist)
The belief that antibiotics reduce fever	It often happens that a patient says, ‘I took antibiotics as soon as I had a slight fever to bring it down; otherwise, my fever would have been much higher by now’” (P.51), (Infectious Disease Specialist)

### Incomplete adherence to treatment regimens

Not completing the full course of treatment is a prevalent issue stemming from a lack of awareness regarding proper medication practices. This behavior significantly contributes to AMR. One participant remarked: *“People*,* due to ignorance*,* stop taking antibiotics after their symptoms improve*,* thinking that continuing the medication is harmful to their body” (P.51).*

### Negative influence of social media

Instead of relying on official sources, individuals often trust incorrect and misleading information disseminated through social media. This misinformation can result in the avoidance of necessary antimicrobials. For instance, one participant noted: *“We face this issue a lot with pregnant women. When a doctor prescribes an antibiotic*,* they ensure it does not negatively affect the fetus. We now have flu vaccinations that we advise pregnant women to use*,* but they still avoid it because they’ve seen negative information on Telegram and similar platforms” (P.39).*

### Unregulated access and storage of antibiotics

The ease of access to antibiotics, both through pharmacies and personal household supplies, emerged as a significant driver of irrational antibiotic use. Participants described how antibiotics are often obtained without prescription and stored at home, contributing to self-medication and inappropriate usage. This phenomenon, sometimes referred to as having a *“home pharmacy”*,* “fridge pharmacy”* or *“personal pharmacy”* enables individuals to initiate treatment without proper medical guidance. One participant explained: *“In Iranian households*,* the fridge often contains various classes of antibiotics*,* which leads individuals to start self-medicating and often using multiple types of antibiotics before they come to the clinic” (P.10).*

A Ministry of Health official emphasized the need for stricter regulations and cultural education to address this issue: *“To better control AMR*,* we need collaboration and planning from the Food and Drug Organization*,* as unfortunately*,* we also witness the overuse of antibiotics in food products at times” (P.3).*

### Lack of commitment to timely medication

Interviewees highlighted a lack of commitment to timely medication as another cultural issue contributing to irrational antimicrobial use. They believe that, in many instances, patients do not adhere to prescribed medication schedules, particularly when repeated doses are required, due to work-related or lifestyle challenges. This non-adherence can lead to suboptimal treatment outcomes and further exacerbate the problem of AMR.

## Service providers’ culture

This theme addresses cultural barriers related to healthcare service delivery that significantly impact AMR management. One major issue is the lack of effective teamwork among health providers, which hinders collaboration in tackling AMR. Additionally, physicians’ fear of reputational damage leads to over-prescribing antibiotics. The culture of medical authority also influences non-compliance with infection control guidelines in hospitals. Furthermore, the lack of professional ethics among services providers contributes to the excessive use of antibiotics. Negative attitudes toward hand hygiene and the unawareness of AMR among veterinarians further exacerbate the issue. Additionally, the preference for new antibiotics, despite existing guidelines, and the neglect of sexually transmitted infections (STIs) by policymakers also pose significant challenges in managing infectious diseases and AMR.

### Weak teamwork in service delivery

Both managers and policymakers, along with operational staff, view the management of AMR as a significant challenge that necessitates collaboration across various levels, from hospitals to policy-making. However, they identify serious deficiencies in teamwork as a barrier to achieving this collaboration. One health ministry manager remarked: *“Coordinating different groups in our society is challenging because teamwork is a cultural weakness. We have experienced this lack of teamwork in old programs like HIV/AIDS. AMR*,* which receives less attention*,* will also suffer if different groups don’t engage in effective teamwork” (P.5).*

### Physicians’ fear of bad reputation and over-prescribing

Interviewees expressed the belief that a considerable amount of medication prescribed, such as excessive prophylaxis during surgeries, is driven by defensive medicine practices aimed at preventing patient complaints and protecting physicians’ reputations. One participant explained:*” Doctors lack trust in hospital infection control and fear being easily penalized. To reduce risk*,* they over-prescribe as a way to compensate for their uncertainty*,* which also helps them maintain patient numbers and avoid complications” (P.3).*

### Negative impact of medical authority on adherence to guidelines

Interviewees identified the culture of medical authority as a significant factor influencing physicians’ adherence to infection control guidelines and standards in hospitals. A manager from an infectious disease specialist association remarked:*“There is a belief among some doctors that they know everything. This mindset is prevalent among doctors. Physicians should always learn from infection control nurses and accept their expertise*,* rather than relying solely on their own judgments” (P.32).*

### Lack of professional ethics

Non-compliance with professional ethics among service providers, including physicians, veterinarians, and pharmacists, contributes to the excessive use of antimicrobials. When economic interests are prioritized over professional ethics, ethical standards can diminish. One participant noted: *“Pharmacists have not yet reached an ideal level of professional ethics. Often*,* pharmacists’ economic interests jeopardize ethical practices*,* and some pharmacists easily ignore regulations and professional ethics” (P.5).*

### Lack of positive attitude toward hand hygiene

Participants noted that a lack of positive attitudes toward hand hygiene among healthcare workers is prevalent, which can contribute to the spread of resistant microorganisms in service environments. One participant remarked: *“There is a hand sanitizing solution above each bed now. Why isn’t it used? Not just by doctors*,* but by all staff. It’s a matter of habit. Knowledge is important*,* but so is attitude and performance. I don’t think it’s a lack of knowledge” (P.41).*

### Unawareness of the problem by veterinarians

Many veterinarians do not recognize the significance of AMR, which contributes to the irrational use of antimicrobials on animal farms. One food safety expert and media activist noted: *“Veterinarians responsible for dairy*,* meat*,* and poultry farms still don’t acknowledge the issue. When I ask why they prescribe so many antibiotics*,* they respond that if they do not*,* clients will go to another vet. They don’t see the problem with resistance” (P.55).*

### Perception of veterinary antimicrobials as less important than human antimicrobials

Managers in the veterinary field expressed concerns about the lower emphasis placed on the quality of veterinary medicines compared to human medicines. An experienced veterinary manager stated: *“Some people in veterinary medicine believe that the sensitivity required for human medicines is greater than for veterinary ones. They argue that since human medicines are for humans*,* there should be more rigor in their production*,* distribution*,* and use. However*,* the impact of veterinary medicines is significant because a large segment of the population is affected” (P.23).*

### Physicians’ preference for new antimicrobials

Similar to the public’s preference for new and expensive antibiotics, some infectious disease specialists believe this issue is also prevalent among physicians. They argue that when new antimicrobials are introduced, some doctors tend to prescribe these medications despite existing guidelines. One participant noted: *“For a simple sore throat that can be treated with a cheap*,* basic drug*,* new-generation* antimicrobials *are often prescribed unnecessarily. These should be reserved for resistant microorganisms where the new antibiotics are our last resort” (P.33).*

### Neglect of sexually transmitted diseases by policymakers

Another significant issue in managing infectious diseases, particularly in human health, is the stigmatization of patients with sexually transmitted infections (STIs). This stigma adversely impacts diagnosis, treatment, follow-up, and data recording for conditions such as tuberculosis and STIs. One participant stated: *“STIs have always been stigmatized and neglected. This affects funding*,* public awareness*,* and patient management. Even in universities*,* they haven’t received adequate attention. We don’t have accurate statistics on these diseases in our country” (P.5).*

Another participant added: *“Patients with STIs often go to private clinics to avoid being noticed*,* and many skip lab tests and follow-ups*,* seeking a quick cure” (P.5).*

## Livestock farmers’ knowledge

This theme highlights the critical role of limited knowledge and awareness among livestock farmers in the misuse of antimicrobials, driven by low literacy levels and poor understanding of proper medication practices.

### Low literacy levels among livestock farmers

Veterinary officials identify low literacy levels among livestock farmers as a key factor contributing to the overuse of antimicrobials and the challenges in implementing AMR policies. Many farmers depend on traditional practices and lack adequate training in animal care, feed management, and biosecurity measures, making it difficult to effectively address issues related to AMR.

### Poor awareness about medications

Farmers often have a limited understanding of medications, their side effects, and appropriate withdrawal periods. One expert noted: *“Farmers view antibiotics as vaccines to boost production” (P.21).*

The lack of education and awareness among farmers significantly contributes to the excessive use of antimicrobials. This issue undermines educational policies and fosters a mindset where farmers perceive medications as tools for profit and increased production. For instance: *“Some farmers see antimicrobials as vaccines that provide immunity to their herds” (P.21). Furthermore*,* “Some farmers use human antibiotics*,* such as human ceftriaxone*,* without proper knowledge about its use and side effects” (P.19).*

## Demographic changes

The theme highlights how demographic shifts—including population growth, aging, and urban marginalization—contribute to the irrational use of antimicrobials. Increased demand for healthcare and animal protein, along with limited access to quality services in underserved areas, amplifies the misuse of antibiotics and exacerbates AMR.

### The role of development and population growth in increased antimicrobial consumption

Economic development and improved conditions in the country have enhanced access to healthcare services and improved health indicators. However, this increased access has also led to the inappropriate and irrational use of antimicrobials, which can accelerate the development of AMR. Additionally, population growth has intensified the demand for animal protein production, resulting in the use of antimicrobials as growth promoters. One participant underscored this concern by stating: *“To ensure food security by 2050*,* we need to increase our animal protein supply by more than 50% in addition to what we currently have… With the increased demand for food*,* the use of antibiotics as growth promoters in livestock*,* poultry*,* and plants has risen” (P.25).*

### Impact of population aging

A health policymaker from the Ministry of Health noted that improved health indicators and increased life expectancy among Iranians have led to a rise in both the use and misuse of antimicrobials. He illustrated this concern with an example involving tuberculosis (TB) patients: *“About 30% of our TB patients are elderly*,* due to the successful control of TB in the country over the past decades. Managing TB in the elderly is particularly challenging because it’s difficult for them to follow all treatment protocols on time and in full” (P.6).*

### Urban marginalization and limited access to adequate services

Urban marginalization and the growth of informal settlements, combined with a lack of health centers and poor sanitary conditions, have restricted access to basic healthcare, adversely affecting the health of marginalized populations. A member of the Rational Drug Use Committee remarked: *“In areas with higher population density*,* naturally*,* there are more visits to doctors. The time that doctors and pharmacists can spend with each patient is reduced*,* leading to irrational prescribing. Moreover*,* many people in these areas don’t visit doctors at all; they resort to self-medication*,* traditional remedies*,* or even various forms of folk healing” (P.7).*

## Discussion

AMR is maintained to be an unavoidable tsunami health systems might encounter in near future. LMICs, including Iran, seem to be more likely to suffer from such an undesirable phenomenon. Sociocultural factors such as demographic changes, incorrect drug culture among the public, cultural challenges among service providers, lack of awareness and knowledge among livestock owners, and the general culture of the public were identified as the underlying contributors to the development of AMR posing challenges to policymaking efforts to combat AMR.

Demographic changes—particularly population growth—have intensified the sharp demand for animal protein, driving the producers to increase the use of antimicrobials in cultivating livestock and poultry in Iran. Globally, it is estimated that antibiotic use for animal food production will rise by up to 67% by 2030 [22]. This situation creates a dilemma between short-term benefits and long-term consequences; while the use of antimicrobials in animal production can enhance livestock output in the short term [23], it ultimately decreases the effectiveness of treatments and contributes to the spread of infections. Consequently, reduced livestock production and trade will result in higher prices for protein sources [24]. Stricter regulations on the use of growth-promoting antimicrobials in livestock and sustainable food production practices are required to minimize the dependence on the antibiotics.

Social inequalities—such as marginalization and limited access to healthcare—were also found to contribute to irrational antimicrobial use. Living conditions play a critical role in infection rates and access to healthcare, as evidenced by the association of treatment-resistant tuberculosis in Europe with factors such as inadequate home ventilation systems and overcrowding in prisons [25]. The high population density in marginalized areas further exacerbates this issue, as a high patient-to-doctor ratio hinders effective patient-provider interactions and examinations, significantly impacting prescription practices [12]. Policymakers should implement equity-focused interventions to enhance services for vulnerable groups, such as marginalized populations, which would also contribute to reduce inappropriate antimicrobial use and mitigate AMR [26]. Gender plays a critical yet often overlooked role in shaping patterns of antimicrobial use and access to care. Women may face unique barriers such as limited financial autonomy, unpaid caregiving responsibilities, and greater exposure to drug-resistant infections due to frequent contact with healthcare systems and menstrual hygiene needs. These factors can delay timely healthcare-seeking and contribute to informal antibiotic use, especially when travel, childcare, or out-of-pocket expenses are involved. Importantly, women are estimated to receive 27% more antibiotics over their lifetime compared to men, underscoring the importance of integrating gender-sensitive strategies into AMR policies and interventions [27]. 

Beyond structural barriers, individual behaviors related to health-seeking and service utilization also emerged as critical factors influencing irrational antimicrobial use. A study conducted in Qom province among students found that over half cited the lack of necessity for a doctor as a primary reason for self-medication [28]. A sociocultural study comparing the prevalence of penicillin-resistant pneumococcus, which was found to be 43% in France and 7% in Germany, revealed that treatment-seeking behaviors are significantly influenced by cultural determinants. In France, individuals sought antibiotics after experiencing symptoms such as coughing and increased mucus production, whereas many Germans considered this unnecessary. Additionally, the doctor-patient relationship and patient pressure for medication in France further impacted antibiotic use, while Germans, due to higher awareness, were less likely to demand antibiotics [29]. These insights suggest the need for culturally tailored awareness campaigns and community-based education to improve understanding of when medical consultation and antibiotic use are appropriate.

Easy and over-the-counter access to antibiotics—contributing to increased self medication—has been reported in several countries, including Syria [30]. This problem is further exacerbated by a widespread preference for new and costly medications among both the general public and healthcare providers. Similarly, in India, physicians often prioritize prescribing imported and higher-cost brands [31]. To address these issues, policies should regulate non-prescription access to antibiotics and encourage evidence-based prescribing practices over brand preferences. In addition, other interventions such as conducting routine inspections of pharmacies, imposing strict penalties for the unauthorized sale of antibiotics, and establishing an online prescription-tracking system to monitor antibiotic dispensing nationwide should be implemented.”

Provider-specific concerns—especially fear of litigation and damage to professional reputation—were found to significantly contribute to the overuse of antimicrobials. This finding is supported by previous research [13]. Furthermore, the issue is not limited to physicians; pharmacists also tend to over-dispense and sell unauthorized medications in response to patient demand [14]. To address these concerns, health authorities should implement enforceable clinical guidelines and promote compliance through monitoring and continuous education.

In addition to individual-level factors, organizational culture—particularly the lack of effective teamwork—was identified as a barrier to appropriate antimicrobial use, which is consistent with previous studies [13, 14]. These studies identified limited interaction and poor collaboration among pharmacists, microbiologists, physicians, and infectious disease specialists as key barriers to rational prescribing in hospital settings. Health systems should strengthen interprofessional collaboration in hospitals through shared protocols and team-based training. However, institutional barriers—such as fragmented governance structures and lack of national intersectoral coordination mechanisms [32]—continue to hinder these collaborative efforts. To overcome these challenges, health authorities should establish centralized AMR governance frameworks, mandate cross-sectoral AMR task forces, and ensure accountability through regular institutional audits.

Extending the discussion on cultural gaps in clinical practice, participants noted that although physicians are aware of the importance of hand hygiene, they do not consistently practice it and lack the necessary commitment to incorporate it into their daily routines [13]. Our findings also suggest that insufficient awareness of the seriousness of the AMR crisis contributes to poor adherence to antimicrobial tiering and the unnecessary prescription of last-resort antibiotics. This concern aligns with other studies that have highlighted physicians’ tendencies to experiment with new drugs and their frequent overprescribing as troubling patterns [14].

To address cultural barriers among service providers, particularly physicians, educational interventions should be implemented starting from medical school and continuing through both initial and ongoing professional development. Previous studies have highlighted that limited access to high-quality educational materials, inadequate continuing education, and the lack of locally adapted clinical guidelines are significant challenges faced by Iranian physicians in rational antimicrobial prescribing [13, 14].

Extending the analysis beyond human healthcare settings, this study found that low literacy and limited awareness among livestock owners regarding antimicrobials contribute to irrational usage patterns. The beliefs, attitudes, and expertise of farmers in disease management significantly influence their antimicrobial use. When a disease occurs, livestock owners assess the costs and benefits of using antimicrobials based on their technical expertise, age, education level, and experience, which in turn influences many of their behaviors [33, 34]. Complementary findings from a recent Iranian study identified key barriers to responsible antimicrobial use in veterinary practice, including inadequate AMR training, weak regulatory oversight, over-the-counter antibiotic sales, and growing client pressure for quick treatments. The use of antibiotics as growth promoters in livestock was also noted as a major concern. These challenges highlight the need for coordinated, multisectoral interventions. Effective planning should address educational, legal, and behavioral factors through policies implemented across individual, social, and institutional levels. Strengthening regulation, reforming veterinary education, and fostering collaboration among universities and regulatory bodies are critical steps to mitigate AMR in the animal health sector and protect public health [34, 35].

Across all sectors, ineffective educational policies were seen as a cross-cutting issue contributing to irrational antimicrobial use. Sweden serves as a model, demonstrating the critical role of public education in raising awareness about the risks of antibiotic misuse, particularly for minor infections. Media campaigns have been instrumental in highlighting both the economic and health-related consequences of AMR. Moreover, simplified prescribing guidelines support rational use among healthcare providers. In addition, Sweden regularly publishes annual antibiotic usage data, updates clinical guidelines online, and provides multilingual educational materials [36]. In contrast, low public awareness in many settings limits policy attention to AMR, representing a missed opportunity for effective intervention [37]. These findings suggest that sustained investment in public education and transparent communication regarding AMR is essential. Enhancing public awareness can not only promote more rational antibiotic use but also foster broader social support and civic engagement, which are critical for advancing and sustaining AMR-related policy actions.

This study is the first to examine sociocultural factors influencing AMR in Iran. The participation of experts from diverse institutions provided a broad and rich perspective on the issue. One limitation of the study was that, due to the demanding policy-making and service responsibilities of some participants, not all were able to review and verify their interview transcripts. This may have slightly limited the depth of the member checking process. Additionally, as with all qualitative research, findings are context-specific and not statistically generalizable. Furthermore, the reliance on self-reported data introduces the potential for social desirability or recall bias, despite measures taken to encourage openness and confidentiality.

## Conclusion

This study highlights that sociocultural factors significantly shape antimicrobial practices and influence the spread of resistant microorganisms across human and animal health systems. These determinants affect stakeholders at multiple levels, from patients and caregivers to healthcare providers and policymakers. It is essential to address this phenomenon not only from a clinical and microbiological perspective but also through an understanding of these social and cultural factors.

Based on participants’ insights, we recommend strengthening public education on rational antimicrobial use, promoting treatment adherence, and enhancing trust in professional diagnoses. Policy priorities should include revising clinical guidelines, improving diagnostic access, and reinforcing oversight mechanisms, particularly through health insurance. Moreover, fostering cross-sectoral collaboration—among physicians, pharmacists, and veterinarians—can help embed antimicrobial stewardship into everyday professional routines. These recommendations should be tailored to the local context and further evaluated for feasibility and effectiveness.

## Electronic supplementary material

Below is the link to the electronic supplementary material.

Supplementary Material 1

Supplementary Material 2

## Data Availability

The datasets used and analyzed during the current study are available from the corresponding author upon reasonable request.
