# Supplementary material for: Sociocultural determinants of antimicrobial resistance in Iran: a qualitative study
Source: BMC Public Health. 2025 Jul 10;25:2425. doi: 10.1186/s12889-025-23361-4 (PMC12243223; doi:10.1186/s12889-025-23361-4)
Supplement: Supplementary file 1 — Supplementary Material 1 [file 12889_2025_23361_MOESM1_ESM.docx]

M / F

- To start with, could you just tell me a little bit about who you are? )Profession / organization(
- Your name and affiliation are completely confidential and will not be published anywhere. You can also not answer a question at your discretion, or interrupt the interview.

1. What do you think are the important social determinants that have influenced Antimicrobial resistance in our country? Please discuss.
2. What do you think are the important cultural determinants that have influenced Antimicrobial resistance in our country? Please discuss.
3. How did these factors affect (decrease or increase) AMR?
4. Have there been any of that have influenced the policies developed to combat AMR?
5. Is there anything else at all that you would like to add?
6. Would it be OK to contact you again?
