# Supplementary material for: Sociocultural determinants of antimicrobial resistance in Iran: a qualitative study
Source: BMC Public Health. 2025 Jul 10;25:2425. doi: 10.1186/s12889-025-23361-4 (PMC12243223; doi:10.1186/s12889-025-23361-4)
Supplement: Supplementary file 2 — Supplementary Material 2 [file 12889_2025_23361_MOESM2_ESM.docx]

Supplementary File 1: COREQ checklist

Consolidated criteria for reporting qualitative studies (COREQ): 32-item checklist

Developed from:

Tong A, Sainsbury P, Craig J. Consolidated criteria for reporting qualitative research (COREQ): a 32-item checklist for interviews and focus groups. International Journal for Quality in Health Care. 2007. Volume 19, Number 6: pp. 349 – 357

| **Item No** | | **Guide Questions/Description** | **Answer** |  |
| --- | --- | --- | --- | --- |
| **Domain 1: Research team and reflexivity** | | | |  |
| **Personal Characteristics** | | | |  |
| 1. Interviewer/ facilitator | | Which author/s conducted the interview or focus group? | Mojtaba Mehtarpour |  |
| 2. Credentials | | What were the researcher’s credentials? E.g., PhD, MD | PhD candidate |  |
| 3. Occupation | | What was their occupation at the time of the study? | PhD student |  |
| 4. Gender | | Was the researcher male or female? | Male |  |
| 5. Experience and training | | What experience or training did the researcher have? | Health policy and qualitative studies |  |
|  | | | |  |
| 6. Relationship established | | Was a relationship established prior to study commencement? | No prior relationship was established between the interviewer and participants before the commencement of the study |  |
| 7. Participant knowledge of the interviewer | | What did the participants know about the researcher? e.g. personal goals, reasons for doing the research? | Participants were informed of the research objectives and the role of the primary researcher through the information sheet and consent form provided prior to the interviews. No personal goals were shared beyond the academic purpose of the study |  |
| 8. Interviewer characteristics | | What characteristics were reported about the interviewer/facilitator? e.g. Bias, assumptions, reasons and interests in the research topic | Participants were provided with information regarding the interviewer’s academic background and the rationale behind conducting the research. |  |
|  | | |  |  |
|  | | |  |  |
| 9. Methodological orientation and Theory | What methodological orientation was stated to underpin the study? e.g. grounded theory, discourse analysis, ethnography, phenomenology, content analysis | This was a qualitative exploratory study. |  |  |
|  | | |  |  |
| 10. Sampling | How were participants selected? e.g., purposive, convenience, consecutive, snowball | Participants were selected using purposive and snowball sampling techniques (methods, sample section, paragraph 1) |  |  |
| 11. Method of approach | How were participants approached? e.g., face-to-face, telephone, mail, email | Face-to-face |  |  |
| 12. Sample size | How many participants were in the study? | 59 interviews conducted (57 participants). |  |  |
| 13. Non-participation Setting | How many people refused to participate or dropped out? Reasons? | One individual declined to participate in the study. The reason for refusal was not explicitly provided, but the individual did not schedule a meeting time |  |  |
| 14. Setting of data collection | Where was the data collected? e.g., home, clinic, workplace | participants’ workplaces |  |  |
| 15. Presence of nonparticipants | Was anyone else present besides the participants and researchers? | No |  |  |
| 16. Description of sample | What are the important characteristics of the sample? e.g. demographic data, date | Job titles are listed in Table 1 at the end of the manuscript. |  |  |
|  | | |  | No |
| 17. Interview guide | Were questions, prompts, and guides provided by the authors? Was it pilot tested? | Yes, the interview guide was developed by the authors based on the study objectives and a review of relevant literature. Five pilot interviews were conducted to refine the guide and enhance its clarity and relevance. |  |  |
| 18. Repeat interviews | Were repeat interviews carried out? If yes, how many? | Yes, two participants were interviewed twice, resulting in a total of 59 interviews |  |  |
| 19. Audio/visual recording | Did the research use audio or visual recording to collect the data? | Yes, most interviews were audio-recorded with participants' consent and transcribed verbatim. |  |  |
| 20. Field notes | Were field notes made during and/or after the interview or focus group? | yes, field notes were made during and immediately after the interviews to capture additional observations and reflections. In cases where audio recording was not feasible, detailed notes were taken to ensure comprehensive data collection. |  |  |
| 21. Duration | What was the duration of the interviews or focus group? | 55 min on average |  |  |
| 22. Data saturation | Was data saturation discussed? | Yes |  |  |
| 23. Transcripts returned | Were transcripts returned to participants for comment and/or correction? | Yes, transcripts were returned to selected participants for comment and/or correction. Some participants provided feedback after reviewing the transcripts, while others, due to their responsibilities, were unable to engage in this process. This limitation has been acknowledged in the discussion section of the manuscript. |  |  |
| **Domain 3: analysis and findings** | | |  |  |
| **Data analysis** | | |  |  |
| 24. Number of data coders | How many data coders coded the data? | One researcher coded the data; codes were reviewed by other team members for consistency |  |  |
| 25. Description of the coding tree | Did the authors provide a description of the coding tree? | Yes, A coding tree was provided in the form of a table summarizing the main themes and sub-themes at the end of the manuscrip |  |  |
| 26. Derivation of themes | Were themes identified in advance or derived from the data? | Themes were primarily derived from the data using an inductive approach, although initial topic areas were informed by the study objectives and relevant literature. |  |  |
| 27. Software | What software, if applicable, was used to manage the data? | MAXQDA 2018 |  |  |
| 28. Participant checking | Did participants provide feedback on the findings? | yes, some participants were invited to review the findings and provided feedback, which was incorporated into the analysis. |  |  |
| Reporting | | |  |  |
| 29. Quotations presented | Were participant quotations presented to illustrate the themes/findings? Was each quotation identified? e.g., participant number | YES |  |  |
| 30. Data and findings consistent | Was there consistency between the data presented and the findings? | YES |  |  |
| 31. Clarity of major themes | Were major themes clearly presented in the findings? | YES |  |  |
| 32. Clarity of minor themes | Is there a description of diverse cases or a discussion of minor themes? | No |  |  |
